# Anatomical Variation and Clinical Diagnosis

**DOI:** 10.3390/diagnostics11020247

**Published:** 2021-02-05

**Authors:** Heather F. Smith

**Affiliations:** 1 Department of Anatomy, College of Graduate Studies, Midwestern University, Glendale, AZ 85308, USA; hsmith@midwestern.edu; Tel.: +1-623-572-3726; 2 School of Human Evolution and Social Change, Arizona State University, Tempe, AZ 85287, USA

**Keywords:** anatomy, anatomical variation, special issues

In the anatomical sciences, it has long been recognized that the human body displays a range of morphological patterns and arrangements, often termed “anatomical variation”. Such variations are quite common and often have no noticeable impact on patient health. However, some variations may cause or contribute to significant medical conditions. An understanding of normal anatomical variation is vital for performing a broad range of surgical treatment modalities and other medical procedures. However, despite their importance for effective diagnosis and treatment, such variations are often overlooked in medical school curricula and clinical practice. Recent advances in imaging techniques and a renewed interest in variation in dissection-based gross anatomy laboratories have facilitated the identification of many such variants. This Special Issue of Diagnostics includes invited and contributed papers that highlight previously under-recognized anatomical variations and discuss them in a clinical context. In particular, the Issue focuses on variants that have specific implications for diagnosis and treatment and explores their potential consequences.

Several studies in this Special Issue present new information that has implications for surgical procedures, especially of the head and neck. Degaga and colleagues described variations in the pneumatization and septation of sphenoid sinus diagnosed using computed tomography (CT) scans, which have implications for trans-sphenoidal surgery [1]. Wang and colleagues evaluated differences in bone parameters at various dental implant sites in the mandible and maxilla [2]. Using cone-beam CT, they found that both cancellous bone density and cortical bone thickness vary by implant site, suggesting that these parameters should be evaluated prior to surgery. Thomas and colleagues presented new information regarding anatomical variations in the recurrent laryngeal nerve [3]. They presented a large-scale cadaveric evaluation and found that extra-laryngeal nerve branches are extremely common, suggesting the need to utilize left-sided approaches for surgical procedures of the neck whenever possible.

Other studies presented implications for surgical and other invasive interventions of the pelvis and extremities. Palomo-Lopez and colleagues documented the anatomic and histological features of the insertion of the extensor digitorum longus tendon on the proximal phalanx of the second toe [4]. They described for the first time the relationship between the tendon, distal nail matrix, and bone, which they explain should be used as reference landmarks during surgical procedures. Granite and colleagues presented a cadaveric study and review of the aberrant obturator artery, which is relevant to pelvic and groin surgeries [5]. Kostov and colleagues evaluated and described a series of anatomical variations in the ureters, pelvic vessels, and nerves that are relevant to the common surgical procedure of pelvic lymphadenectomy [6]. Finally, in a potentially revolutionary study, Garner, Plochocki, Hall, and colleagues describe how anatomical variation affects the potential for iatrogenic damage during episiotomy [7]. They discovered that episiotomies involving a midline incision resulted in a reduced risk of injury to nerves, erectile tissue, muscles and glands of the region than those involving mediolateral incisions. In the upper extremity, Sawyer and colleagues clarified the branching and innervation patterns of the radial nerve in the forearm [8]. They revealed significant variability in the posterior forearm, including occasional innervation of the brachialis and the superficial radial branch periodically innervating the brachioradialis.

A number of studies revealed anatomical and developmental bases for various pathologies and clinical presentations. Lee and colleagues described longitudinal changes in the tibiofibular relationship in patients with hereditary multiple exostoses [9]. A previously undescribed relationship was revealed in which the tibia grows relatively faster than the fibula, resulting in valgus angulation. Mirande and colleagues described two cases of retroesophageal aberrant right subclavian artery (ARSA) and demonstrated the enlarged size of the vessel relative to a normal sample [10]. As patients with this condition may present with dysphagia or be asymptomatic, they suggest that the relative size of the ARSA may dictate the severity of the associated symptomatology. Dyches and colleagues describe the acute angle of the celiac trunk in individuals with median arcuate ligament syndrome (MALS), thus explaining the mechanism of arterial compression in this condition [11]. They also described how, contrary to popular belief, the origin of the celiac trunk is not more superiorly positioned in patients with MALS compared to the general population. Grande-del-Arco and colleagues described the effect of tube angulation on the distortion of the metatarsal I head shape and clarified the correct shape of the metatarsal head in anatomical dissection [12].

Finally, Petto and colleagues presented a case of a rare anatomical variant, musculus sternalis [13]. They describe the unusual anatomy and explain how its finding in a gross anatomical teaching laboratory setting informed healthcare students about the importance of appreciating variation in diagnosis and treatment.

These papers present a variety of intriguing, clinically relevant anatomic variants, and explain their implications for surgical interventions, developmental understanding, and diagnoses. They emphasize the wealth of information that is still unknown about human anatomy. These studies also highlight the important contributions of body donors to medical education and the advancement of medical treatments, as many studies were conducted using cadaveric specimens. It is our sincere hope that the information in this Special Issue will be applied to advancing the diagnosis and treatment of patients with anatomic variants.
